# The relationship between healthcare service provision models and patient experience

**DOI:** 10.1108/JHOM-06-2021-0242

**Published:** 2021-12-24

**Authors:** Sabina De Rosis, Chiara Barchielli, Milena Vainieri, Nicola Bellé

**Affiliations:** Institute of Management and Department EMbeDS, Scuola Superiore di Studi Universitari e di Perfezionamento Sant'Anna , Pisa, Italy

**Keywords:** Healthcare provision models, Patient experience, PREMs, Nursing care models, Excellence in user experience, Quality improvement

## Abstract

**Purpose:**

User experience is key for measuring and improving the quality of services, especially in high personal and relation-intensive sectors, such as healthcare. However, evidence on whether and how the organizational model of healthcare service delivery can affect the patient experience is at an early stage. This study investigates the relationship between healthcare service provision models and patient experience by focusing on the nursing care delivery.

**Design/methodology/approach:**

65 nurses' coordinators were involved to map the nursing models adopted in the healthcare organizations of in an Italian region, Tuscany. This dataset was merged with patient experience measures reported by 9,393 individuals discharged by the same organizations and collected through a Patient-Reported Experience Measures Observatory. The authors run a series of logistic regression models to test the relationships among variables.

**Findings:**

Patients appreciate those characteristics of care delivery related to a specific professional nurse. Having someone who is in charge of the patient, both the reference nurse and the supervisor, makes a real difference. Purely organizational features, for instance those referring to the team working, do not significantly predict an excellent experience with healthcare services.

**Research limitations/implications:**

Different features referring to different nursing models make the difference in producing an excellent user experience with the service.

**Practical implications:**

These findings can support managers and practitioners in taking decisions on the service delivery models to adopt. Instead of applying monolithic pure models, mixing features of different models into a hybrid one seems more effective in meeting users' expectations.

**Originality/value:**

This is one of the first studies on the relationship between provision models of high-contact and relational-intensive services (the healthcare services) and users' experience. This research contributes to the literature on healthcare service management suggesting to acknowledge the importance of hybridization of features from different, purely theoretical service delivery models, in order to fit with providers' practice and users' expectations.

**Highlights:**

- This is one of the first studies on the relationship between provision models of nursing care and patient experience.
- Healthcare services' users appreciate service delivery characteristics identified with “be cared by,” or in other words with having a reference nurse.
- Nursing models' features that relate to the organizations and that providers tend to judge as professionalizing and evolutive, such as team working, appear not key in relation to patient experience.
- Pure models of service delivery are theoretically useful, but hybrid models can better meet users' expectations.

## Introduction

**Figure 1:**
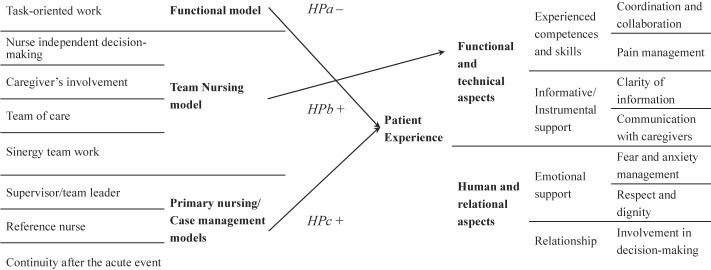
Brief representation of the hypotheses to be tested in the research

Services are, by their nature, always experienced ( Carbone and Haeckel, 1994 ), including healthcare services delivered by public providers: “ *In no other service is managing the ‘customer experience’ more important than in healthcare* ” ( Berry, 2019 ). The patient experience with healthcare services is a key indicator of quality and outcome, and a key aspect to manage for practitioners ( De Rosis *et al.* , 2019 ), contributing to measure several aspects of the quality in healthcare including person-centeredness ( Baker, 2001 ). How the patient experiences safety, respect, dignity and kindness is a key metric to determine the person-centeredness of healthcare systems, and a tool to innovate and co-produce the future developments of the same systems ( Anderson *et al.* , 2018 ; De Rosis *et al.* , 2019 ). Focusing on patient experience is part of an overall strategy for improving performance and outcomes, such as reducing hospital readmissions ( Anderson, 2021 ). According to Anderson *et al.* (2018) , the patient participation can realize the patient centricity as the health services' delivery approach, by co-creating value in healthcare (see Figure 1 ).

Despite its importance, research on patient experience remains fragmented ( Jain *et al.* , 2017 ). People's perception of patient-centered care is associated with the need for the work in teams of healthcare professionals, for respectful and compassionate care, and for patient-involvement, engagement empowerment ( Gogovor *et al.* , 2019 ; Jaensch *et al.* , 2019 ). Nevertheless, there is little evidence on whether and how the organizational model of healthcare service can affect the patient experience. This is true also, specifically, with regards to nursing care. The nurses are among the front-line professionals who first meet patients in different healthcare settings, and spend most of their time with them, especially during their hospital stay. Nurses have a significant impact on the quality of healthcare; and patients have specific expectations from nurses: medical knowledge, competences, safety, trust and proper communication ( Wasik, 2020 ). At the same time, the patient experience has been identified as a key aspect to consider in defining evidence-based practices and models of nursing care ( Schaffer *et al.* , 2013 ).

The aim of this research is to investigate whether and how different organizations of the nurses' work, tasks and interactions with patients and caregivers affect the patient experience, with the final goal to identify nurse-sensitive aspects of the patient experience, and the characteristics of the nursing care models that can finally improve the latter.

## Theoretical framework and hypotheses

In the following sections, we will first illustrate each of the two constructs that we measure, patient experience and organizational models, and then formulate our hypotheses.

### Patient experience

Patient experience is a multidimensional construct, which includes cognitive, emotional and sensorial aspects, as well as behavioral reactions to external stimuli, such as human interaction or facility features ( Berry *et al.* , 2006 ). According to Berry *et al.* (2006) , perceptions of an experience are based on technical performance of the service, its tangible aspects and relational and behavioral aspects linked with interactions with service providers ( Berry *et al.* , 2006 ). The relative importance of the above-cited dimensions can change according to the intensity of some characteristics of the service itself. A recent study on hospital case management services, which assessed the overall quality of and patient satisfaction with the services, using patient expectations and perception, showed that the intangible aspects are the most important predictors of a positive patient experience ( Perera and Dabney, 2020 ).

In fact, healthcare services are highly personal and relation-intensive ( Hausman, 2004 ; Berry and Bendapudi, 2007 ). Patients mostly describe experiences with healthcare by referring to the healthcare professionals' behavior rather than their technical expertise ( Berry and Bendapudi, 2007 ). This aspect is also linked to the level of patient involvement, communication and education by the healthcare personnel ( Hausman, 2004 ). These activities are not meant as merely informing patients, which conversely can be defined as an instrumental support. They are key, considering that objectification, standardization and commodification in healthcare can lead professionals to reduce patients to their disease with little focus on the actual person behind the illness ( Timmermans and Almeling, 2009 ). Scholars identified informational support as one of the two components of social support seeking behaviors of people for coping with stressful encounters; the other component is the emotional support ( Stanisławski, 2019 ). Relational and emotional components of the social support are relevant in healthcare, where interpersonal interactions can greatly affect the patient experience and satisfaction ( Hausman, 2004 ). They can create a positive experience and maintain a positive relationship over time ( Jain *et al.* , 2017 ). This is particularly important considering that people would not experience healthcare services, but they could need to. Previous research has emphasized the role of compassion and communication in caring for patients, which highly impact the patient experience with nursing care ( Jakimowicz *et al.* , 2015 ). In this research, we classified the abovementioned dimensions of patient experience using the Berry and colleagues' categorization in technical and human aspects of service delivery ( Bendapudi *et al.* , 2006 ). A summary of the dimensions, with some of their aspects and proxies, are presented in Table A1 ( Appendix ).

The patient experience is an important indicator of care quality, and it is also considered an outcome of healthcare services ( Abdel Maqsood *et al.* , 2012 ).

During hospitalization, the share of nursing care is dominant. Therefore, a positive experience with nursing care delivery represents one of the healthcare systems' goals to achieve, also considering that patient satisfaction is positive associated with the patient propensity to follow medical and nursing prescriptions after their hospital stay ( Buchanan *et al.* , 2015 ). It is also a proxy for satisfaction with the whole healthcare service ( O'Connell *et al.* , 1999 ). Indeed, separating the patient satisfaction with nursing care from the overall experience with the hospital care is challenging. Despite the attempt to identify specific elements of patients' satisfaction with nursing care, no consensus has been reached in literature ( Abdel Maqsood *et al.* , 2012 ). Moreover, little research has been devoted to the relations between nursing organizational models and patient perception of care quality. Research has mainly focused on the relationship between nurse staffing levels and patient outcomes ( Hall and Doran, n.d. ; Burston *et al.* , 2014 ; Dubois *et al.* , 2013 ; Griffiths *et al.* , 2016 ), as well as patient experience with the care process ( Bruyneel *et al.* , 2015 ; Aiken *et al.* , 2018 ; Griffiths *et al.* , 2013 ), showing a relationship of patient satisfaction with nurses' job satisfaction and staffing level ( Kvist *et al.* , 2014 ). Some studies report patient satisfaction or experience with nursing care without exploring whether and how different features of the nursing care delivery differently affect the patient experience ( Bruyneel *et al.* , 2015 ; Aiken *et al.* , 2018 ).

In addition, the definition of nurses' work, tasks and responsibilities has changed over time, as a result of organizations' development, evidence availability, and increasing expectations of patients. Different models of nursing have been developed and applied, by gaining attention to one rather to another characteristic of the nursing care delivery.

### Nursing models

Nursing care delivery or organizational models provide guidance for professionals in achieving clinical objectives and serve the purpose of evaluating the outcomes ( Edward, 2015 ). According to Dubois *et al.* (2012) , the organizational model is the result of key resources and process features that defines how delivering nursing care at the unit level. The way these elements (e.g. nurse to patient ratio, staffing, grouping of patients by pathology) are combined defines the ultimate goal of nursing care and differentiates professional practice models. Hutchinson *et al.* (2014) identified seven domains characterize nursing models: “ *autonomous or nurse-led extended clinical practice, improving systems of care, developing the practice of others, developing/delivering educational programs/activities, nursing research/scholarship, leadership external to the organization and administering programs, budgets, and personnel* *.* ”

According to the literature on the traditional models of patient care ( Tiedeman and Lookinland, 2004 ), in this study three main delivery models have been identified, with eight specific features, characterizing different ways of organizing processes, tasks and responsibilities of nurses ( Appendix – Table A2 ).

In the functional nursing (FN) model, nursing care is organized and provided by nurses around specific tasks ( Davis, 1993 ). For instance, a nurse is in charge of providing medications, another one is in charge of providing personal care, and this is meant for every single patient that is in the ward at a given time, regardless different patient's characteristics and needs. Nurses are totally activity-oriented and have to report to the head nurse ( Berry and Metcalf, 1986). This organizational model is particularly effective when in need of performing a vast variety of tasks in a short time or when there is a scarcity of personnel. In fact, it developed during the Second World War. From the patient perspective, care is delivered by different nurses, with a clear fragmentation of care.

Conversely, the team nursing (TN) model encompasses the presence of a group of nurses with different skills and competences, who are coordinated by a team leader and are in charge of caring specific groups of patients. This model was born in the 70s' United States of America, characterized by a lack of nursing staff. The team provides a total care service for a given patient, including the various tasks that are provided separately in the FN model. A key feature of the TN model is the collaborative work: the patients' care is a group effort, under the responsibility of a team leader, following a nursing care plan and a process of identification, planning, implementation and evaluation of care according to different patients' needs ( Davis, 1993 ).

Primary nursing (PN) is an organizational model characterized by the assistance continuity. Care delivery is organized around the patient. The primary nurse is responsible for a given number of patients and for each of them draws a care plan, based on a previous assessment and adjusted over time. Other nurses can assist the patients, following the care plan. In this model, the nurse-patient relationship presupposes a great availability of staff. Nursing Case Management (CM) is a collaborative approach delivering care interventions to a specific group of patients, and it is meant to follow, assist and coordinate interventions throughout the continuum of healthcare services ( White and Hall, 2006 ). The case manager nurse operates in autonomy and manages patient's needs, but also operates a function of costs containments, avoiding duplications in interventions. PN and CM models share a number of common features like the reference nurse and the responsibility for the entire spectrum of care for the entire duration of the hospital stay and beyond ( Tiedeman and Lookinland, 2004 ).

### The relationship between patient experience and nursing models

As anticipated, there is growing evidence that nursing care organization and provision are critical factors determining patient outcomes in hospitals ( Kurtzman, 2010 ). Moreover, nurses seem the most supportive of the patient-centered care approach among the different healthcare professions ( Gogovor *et al.* , 2019 ). Thus, examining the organization of the nurses' work in relation to the patient perception can provide valuable insights on those aspects that make a difference in the patient experience with care.

Because to the best of our knowledge there is a lack of empirical studies on the relationships between patient experience and its determinant factors related to the nursing models, this research investigates the associations between patient experience, and the characteristics of the nursing care delivery reported by professionals. Based on our abovementioned theoretical framework, we argued that:

1. The task oriented (FN model) is negatively associated with both technical aspects, and human aspects of the patient experience.
2. The characteristics of the TN model are positively associated with the technical aspects of patient experience.
3. The characteristics of the PN/CM model are positively associated with both human and technical aspects of the patient experience.

Since the FN model is task-oriented, we expect that it is overall negatively associated with the patient experience. In this study, the technical aspects of patient experience are not referring to single tasks, but to coordination and collaboration (HP5), pain management (HP6), information at discharge (HP9 and HP10), and clear answers of nurses to patients (HP8) and communication with caregivers (HP7). Since the FN model can produce a fragmented care, we hypothesized that this model may negatively affect the patient perception of the abovementioned aspects. In addition, we expect that a task-oriented work negatively predicts the human aspects of service delivery, since a single patient is cared by several nurses, namely fear and anxiety management (HP1), respect and dignity (HP2 and HP3) and patient involvement in decision making (HP4).

We hypothesize that the informative and instrumental support (HP7–HP10) would be positively affected by the features of the TN model, because of the easiness of communication in coordinated teams. Similarly, the personnel competences and skills would be better evaluated by patients, particularly in relation to coordination and collaboration among members of a structured team (HP5).

Finally, we drew the hypothesis of a positive association of the PN/CM model and all items using for exploring the patient experience in this study, because of the presence of a reference/case manager nurse who can create the premises of a better nurse-patient interaction and communication (HP1–HP4, HP7–HP10); and because of the presence of a team leader who coordinates the work of nurses (HP5 and HP6). We also tested the effect of each nursing models' feature on the satisfaction measures: willingness-to-recommend (HP9) and overall evaluation of care (HP10), by hypothesizing that the feature of the TN model is the only one negatively affecting the satisfaction of patients.

**Figure A1:**
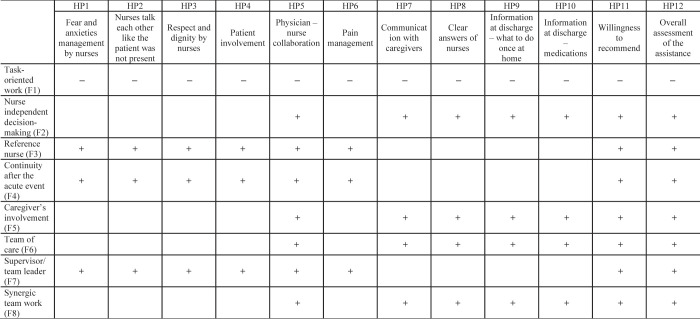
Scheme of the hypotheses to be tested

Figure A1 in Appendix reports a scheme of the above mentioned hypotheses.

## Method

This study uses both qualitative and quantitative data, from two primary sources: cross-sectional Patient-Reported Experience Measures (PREMs) sourced from a permanent Observatory on patient experience in Tuscany Region (Italy), and data from a mapping of the nursing practice models, performed in Tuscan Local Health Authorities and Teaching Hospitals.

### Data type, sources and collection instruments

PREMs are collected by the means of questionnaires measuring patients' perceptions of their experience whilst receiving care in hospital ( Nuti, 2008 ; De Rosis *et al.* , 2020 ). The PREMs questionnaire is developed from the Picker Institute questionnaire ( Jenkinson *et al.* , 2002 ), widely used and validated in several countries. The survey is digitally administered to discharged patients and includes standard questions on patient experience of hospitalization, covering the key dimensions abovementioned. The full questionnaire is available in Appendix of De Rosis *et al.* (2020) .

This paper analyses PREMs collected in the Tuscan hospitals from March 2018, by focusing on specific aspects of the patient experience with hospitalization. Table A3 in Appendix reports the single items and the related scales. Several studies have found that multi-items scale does not necessary outperform single-item scales under certain circumstances and that, for the many constructs that consist of a concrete singular object, single-item measures can or should be used ( Bergkvist and Rossiter, 2007 ; Gardner *et al.* , 1998 ). Previous research on patient-reported experience measures has also used both multiple-item constructs and single items ( Bjertnaes *et al.* , 2012 ). Thus, we intentionally choose to investigate each single item of experience, instead of the aggregate dimensions or aspects of experience with services, since we are interested in understanding what effect each characteristic of the organizational models can have on the most granular level of detail captured by the experience survey. Moreover, the experience constructs used in this research are composite constructs; while we want to specifically test, where possible, the impact of nursing care organizational models on the care experience.

As reported in Table A3 ( Appendix ), some variables measuring patient experience directly refer to the nursing care delivery, to obtain a specific measure of the patient perception of nurses' contribution to their experience. In addition, because the nursing care can influence the overall care experience, the other items refer to the care provided by healthcare professionals in general, and to the patient satisfaction, namely willingness-to-recommend (WtR) and overall satisfaction with the care service.

Data for determining the nursing practice model adopted in each hospital ward were gathered from a mapping performed in three Local Health Authorities (LHAs), three Teaching Hospitals, and one mono-specialist hospital in Tuscany. The mapping involved 65 nurses' coordinators, who filled a questionnaire in June 2019 ( Appendix – Table A4 ). They had to reflect on the internal organization of the nursing care delivery adopted in their departments, and report what features mainly represent it. Specifically, they were asked to indicate in what percentage every characteristic was present in their daily organization of work. Each item encompassed a scale on 4 levels, where 1 indicates that the specific feature is adopted for less than 30%, 2 for an adoption between 30 and 50%, 3 for 50–70%, 4 for more than 70%. Each nursing model was computed as an index given by the mean of the values of their characteristics as listed in Table A4 ( Appendix ).

### Method of data analysis

At the time of the nursing model mapping, the PREMs Observatory was ongoing in the three THs, in the mono-specialist hospital and in two out of the three LHAs. Therefore, first, PREMs were selected on the base of the date of the nursing models mapping (March 2018–June 2019), and of wards/departments involved in both studies, to have the same departments in the two datasets.

Second, the percentage of presence of each nursing model feature, as emerged in the nursing models mapping, was merged to the PREMs dataset, as new variables characterizing the ward of discharge of each individual patient. Using the merged dataset, we run descriptive analyses, for exploring the patient perception of their experience with the hospital care by the means of the distribution of the different variables ( Appendix – Table A3 ).

The final step included a preliminary correlation analysis using all abovementioned variables. Therefore, 12 logistic regression models were run to test the relationships among variables. Each model had, as the dependent variable, one specific aspect of the patient experience ( Appendix – Table A3 ); and as independent variables, the characteristics of the nursing models ( Appendix – Table A4 ). Due to the usual positive skewed distribution of patient evaluation ( Munro *et al.* , 1994 ), also emphasized by the digital survey, the dependent variables were recoded as binary, to perform linear regression logistic models: one stands for the most positive option and zero represents all other options of answer. For instance, if a patient reported as “always clear” the nurses' answers, the value of the variable was re-coded as one, while all other options (“often – sometimes – rarely – never clear”) were recoded as zero. Such dichotomization provides an intuitive interpretation of the results, and is also more robust to the censoring ( Ko *et al.* , 2019 ). The models were used to test whether the nursing models' features made the difference in building an excellent patient experience. Additional potential confounding factors were added to the models, namely: patient age (continuous variable), sex (dummy variable), educational level (categorical) variable, perceived health status (categorical variable), access from the emergency department (ED) (dummy variable), support of someone in filling-in the questionnaire (dummy variable). Results are presented using odd ratios (ORs). All necessary specification tests have been checked and the overall fitness of the model was also verified by using the value of Pearson Chi-square test. The statistical significance was set at *p* < 0.05.

## Results

### Nursing models: descriptive results

The mapping of the nursing models shows that there is not a pure model put in practice in Tuscany, rather a hybridization between the three models described in the introduction ( Table 1 ). The functional model is almost always present, but, overall, it represents less than 30% of the current practice. The TN model is near to the range 30%–50%. From the correlation analysis among the three models, it emerged that the functional model is negatively correlated with the TN model (−0.19; *p* < 0.001), while it is not significantly correlated with the PN/CM model.

The distribution of the various characteristics of the nursing models are reported in Table A5 ( Appendix ), while the detailed results of the correlation analysis (1) among all features and (2) with the nursing models are reported in Appendix – Table A6 .

### PREMS: descriptive results

Selected data from PREMs refer to 9,393 respondents, both patients who responded on their own ( *n* . 7,131; 75.9%) and patients who have been helped by someone in responding ( *n* . 2,262; 24.1%) ( Table 2 ). There is a slight majority of males among the respondent patients (51.85%).

The low educational level counts around a half of the respondents ( *n* . 4,550; 48.5%); medium and high educational levels represent respectively 35.7 and 15.8%. People helping in filling the questionnaire have mainly a medium educational level (48.5%, *n* . 1,043). A half of the respondents reported to be chronic patients ( *n* . 4,402; 49.5%). Among these latter, more than a half claimed to have been hospitalized for the chronic diseases they suffer of ( *n* . 2,601, 56%) (not in Table 2 ).

Table A7 reports patient experience measures, by showing mean, standard deviation and percentage of responses. Considering the abovementioned positive skewed distribution, PREMs with scales from 1 to 5 were re-coded to 3-level variables, to emphasize the difference in the patient evaluation. Values 4–5 were coded as “excellent and good rating,” while values 1–2 were coded as “week and poor rating” (see “Options of answers” in Table 3 ). Overall, the results show that the experience reported by patients was very good ( Table A8 – Appendix ).

### PREMS and nursing features: regression models' results

Table 3 shows the results for the 12 regression models performed for each experience dichotomic variables, to identify which organizational feature of the nursing models (independent variables) was positive associated with an optimal experience with the healthcare service (dependent variable). It includes the value of ORs and the level of significance ( *p* -value). In the following lines, we describe the effect of each independent variable on the probability of an excellent experience, other things being equal.

The analysis shows that, as age increases and educational level decreases, the likelihood of an excellent patient experience also increases ( *p* < 0.001), with respect to almost all its aspects, and with a stronger effect of the first one. The age effect is always significant, while the educational level is not significant with respect only to pain management, information at discharge on medications and overall evaluation of care.

A negative perception of health status and hospitalization following ED access are negatively related to an excellent experience, with a very high significance ( *p* < 0.001). In both cases, the effect of these two variables is particularly high. On the contrary, the more positive the perception of one's health status, higher the probability of excellent evaluation with an effect on OR ranging from 111% (WtR) to 3% (patient involvement). Hospital access through ED is always significantly, and mostly negatively, associated with respect to excellent evaluations. The OR variation was more than 50% for patient involvement, and communication with caregivers, and around −80% for overall assessment of care, clear information at discharge, and nurse-physicians collaboration. Only with respect to the information clarity at discharge regarding medications, the access through ED has a lower significance ( *p* = 0.025).

Being a man is significantly and positively related ( *p* < 0.001) in the evaluations of patients' dignity respect, and nurses-physicians collaboration (both slightly more than 20%). The significance of the effect is lower when it comes to WtR (25%, *p* = 0.01) and overall care assessment (10%, *p* = 0.043).

If the patient responds alone, the probability that the assessments of the experience are excellent are always and significantly higher ( *p* < 0.001), especially with regards to the WtR (539%) and to the clarity of the answers received by nurses (211%). In all other cases, the factor change variation the variation is around 100–150%, except for the case of nurses talking as the patient was not there, where the factor change is 77%.

**Figure A2:**
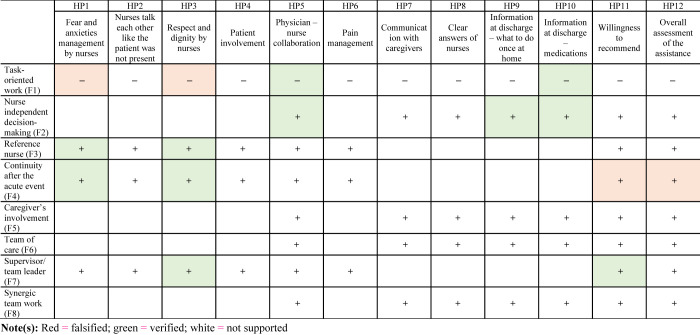
Results of the hypotheses tested with the regression models. A color version of this figure is available online

In Figure A2 ( Appendix ), the colors indicate if the hypotheses were verified or falsified by the results of the regression models. While in the following lines, the results are presented by considering the effect of the characteristics of each nursing organizational model on the various patient-reported experience and satisfaction measures. No nursing organizational models' feature appears to make a difference in the perception of the quality of communication with caregivers (informative/instrumental support), including the TN model's characteristic “caregivers' involvement” (F5), as well as in the experience of the pain management (experienced competences and skills).

The characteristic of the functional model does not negatively affect all aspects of the patient experience. “Task-oriented work” (F1) is positively related to patients' experience with fears and anxieties management (9%, *p* = 0.016) and the respect of the patient dignity (11%, *p* = 0.01). Conversely, it is negatively associated with the perception of a good physicians-nurses collaboration, and with the quality of information received at discharge (respectively −10% e −9%).

The two features of the TN model that do not play a relevant role in making excellent the patient experience are the organization of a defined team of care (F6), and the synergistic work (F8). Additionally, the more nurses decide independently (F2), the better is the patient assessment: a positive variation in OR is registered in the experience of an excellent respect of patient's dignity (14% “Talk like the patient was not present,” and 10% “Respect and dignity,” *p* = 0.004), and in the information clarity at discharge (14% on “what to do once at home,” *p* = 0.009; 19% on “medications,” *p* < 0.001). The association is also positive with respect to the physicians-nurses collaboration (9%, *p* = 0.015). The caregivers' involvement (F5) results positively associated with the way in which nurses managed patients' fears and anxieties (8%, *p* = 0.036), and the patient involvement in decision-making (11%, *p* < 0.001). Results only partially confirm the initial hypotheses, by reporting a more general impact on patients' experience conveyed by the nurses' organization in team.

Finally, by looking transversally at the regression models' results related to the features of the PN/CM model, counter-intuitively enough, the feature “continuity after acute event” (F4) has a negative effect on many dimensions of the patient experience: emotional support by nurses (fears and anxieties management −14%, *p* = 0.008; respect and dignity, −22%, *p* < 0.001); information support (clarity of responses of nurses, −16%, *p* = 0.002); satisfaction (WtR −19%, *p* = 0.028; overall assessment, −14%, *p* = 0.003). Having a reference nurse (F3) positively affects the management of fears and anxieties by nurses (9%, *p* = 0.001), the evaluation of respect and dignity (11%, *p* = 0.001) and the overall assessment of the hospital care (4%, *p* = 0.08). On the other hand, it has a negative effect on the information clarity at discharge on medication (−8%, *p* = 0.004). The presence of a designated supervisor or team leader (F7) is positively associated with the evaluation of respect and dignity (10%, *p* = 0.005), clarity of nurses' answers (8%, *p* = 0.005) and WtR (11%, *p* = 0.023).

## Discussion and practice implications

This study breaks new ground in at least two ways. It measures the patient experience with hospitalization with reference both to the specific contribution of nurses, and to the overall patient experience and satisfaction with care provided by the healthcare staff in general. Despite prior research has raised the possibility that the way in which the nursing care is delivered can affect the patient experience, this is the first study to measure and analyze the relationship between each specific characteristic of the nursing care models, and the patient experience. The results of the nursing models' mapping support the choice of considering the nursing models' characteristics in the analyses as the most important elements, instead of seeking a pure model as in previous research on patient outcomes ( Dubois *et al.* , 2013 ). A recent Italian study has shown how the organizational characteristics of nursing care practice, leadership style, and nursing staff behavior affect patients' perception of the nursing care quality ( Zaghini *et al.* , 2020 ). To this respect, the findings of this study support the idea that the nursing care delivery not only has an impact on the patient experience with nurses, but it is also able to affect the overall experience and satisfaction of patients with the hospitalization service, and their perception of care provided by the different healthcare professionals.

Larsson and colleagues' work ( 2007 ) highlights how the most typical aspects of nursing care are related, from the patient's point of view, to participation (i.e. atmosphere of kindness and helpfulness, respect, dignity), to the emotional support and to the ability to cooperate in a team. Both emotional and instrumental support are of great importance in order to help them in facing and managing the situation of stress caused by illness and hospitalization experiences ( Stanisławski, 2019 ).

Considering the emotional aspects of the patient experience, it emerged that this latter is positively affected by various features of the three nursing models. Task-oriented work, nurse independent decision-making, reference nurse and team supervisor are positive associated with an excellent patient experience in terms of respect and dignity, as well as with other aspects of the emotional support or, as defined by Larsson *et al.* (2007) , the atmosphere of kindness and compassion created by nurses, which produce an emotional response in the patients. As reported by Baret *et al.* (2018) , nurses are always accountable for care provided to patients, regardless of environmental and organizational factors for which they are not in control of, such as stressful and cost constrained environments. Nevertheless, this study shows that the way in which the nursing care is organized matters: in particular, only having someone who is in charge of the patient, both the reference nurse and the supervisor or team leader, makes a real difference. An excellent patient experience is associated with the characteristics of the nursing organizational models referring to the presence of a nurse who is individually, or as a supervisor, responsible of the patient care. This can be read as the patient appreciation for having someone clearly taking charge of his/her care. The reference nurse and the supervisor introduce themselves to the patient as the one “in charge of them” and make explicit the fact that the patient must refer to them. This particular element can reduce the uncertainty perceived by the patient ( Feo *et al.* , 2017 ). The appreciation of these aspects of nursing care delivery model can be also explained also by the fact that these aspects have a significant common ground with the attributes of the patient-centered care, in particular if compared with the care delivered by other healthcare professionals' groups ( Gogovor *et al.* , 2019 ).

Task-oriented nurses' work also resulted positively associated with the patient experience for what concerns the emotional dimension. Although this result can appear surprising, this is an unavoidable characteristic of the nurses' work. Nevertheless, the more this feature characterizes the nurses' work organization, the more nurses are likely to be demotivated and unsatisfied ( Tappen, 1994 ). Moreover, the negative association between task-oriented work and doctors-nurses collaboration and quality of information, in particular at discharge, is confirmed by the results here presented.

The concordance in the information provided to patient has been acknowledged as a key aspect of the nursing care ( Larsson *et al.* , 2007 ). The instrumental or informative support is crucial to reduce stress and anxieties, and to enable them to autonomously manage health issue after discharge ( Lemos *et al.* , 2009 ). The results of this study underline how the nurses' autonomy in taking decisions is the sole nursing care models' feature that resulted positively associated with an excellent experience with the clarity of information at discharge. This may be linked to the fact that the nurse established a clear relationship from the beginning and the consequentiality of the given instruction is clearer to the patient.

On the contrary, having a reference nurse improves the emotional support but does not make a difference in relation to the informative support at discharge. This can be explained by the possibility that the discharge could have happen during the absence of the reference nurse, and that information provided by another nurse could have been not concordant with that given by the reference nurse.

This study also showed that the caregiver's involvement as a feature of nurses' work does not affect the patient perception of the easiness of communication between the hospital staff and the caregivers; while it is a key aspect in the experience of patient involvement, nurses' pain management and respect of patients' dignity. The effect of these practices should be additionally studied, since literature mostly focused on caregivers' involvement in the patient transition from hospital to home, or another healthcare facility/setting ( Hahn-Goldberg *et al.* , 2018 ; Murray *et al.* , 2019 ), and in the caregivers' attention for pain management, in particular with respect to fragile patients ( Juarez and Ferrell, 1996 ). Additional research could be undertaken to compare experience of different groups of patients, or discharged from specific specialties or wards (i.e. geriatrics).

A very interesting result is that the characteristics of nursing care models not referring to individual nurses' specific behaviors or organization of work, that is to say those features that mostly refer to the organization of teams, are not significantly or positively associated with the patient experience. In particular, while the features “team of care” and “synergistic teamwork” are not significantly associated with the patient experience, “continuity after the acute event” is negatively associated with some aspect of experience related to emotional and informative support, as well as to the patient satisfaction. These organizational features seem to be transparent from the patient point of view, not affecting any aspect of the patient experience here investigated. However, their importance is self-evident in the delivery of high-quality care to patients. Further research should be undertaken in this area, bearing in mind that patients and nurse can have different, sometimes contrasting, opinions on what high-quality care is ( Greenhalgh *et al.* , 1998 ).

Given these results, it could be argued that improving the patient experience may affect the hospitals' “productivity”. In other words, for producing better experiential outcomes, a clear and evident presence of nurses nearby the patients' bed can require additional resources; or a more accurate and person-centered communication aimed at making evident and understandable aspects of the nursing care that are currently “transparent” (such as the nursing models' features related to the team) can encompass additional nurses' time with patients and the improvement of their communication and soft skills. On the contrary, a good balance between the technical quality provided by healthcare professionals and the experiential quality as perceived by patients are not in contrast and do not diminish the productivity of the healthcare organizations; on the contrary, a higher overall quality that combines these two quality dimensions enhance their performance ( Anderson and Smith, 2018 ). “There is the potential to spend ample time with patients and still be financially sustainable” ( Smith *et al.* , 2020 ). The results of this study suggest that managers should find a balance between the nursing care models' characteristics that assure a good technical care and a constant attention to experiential quality of patients. Of course, it remains crucial to understand the patient preferences' heterogeneity, to customize the practices of interaction and communication, and their implications in terms of technical and experiential outcomes ( Pham *et al.* , 2021 ).

Overall, the findings of this study confirm that there is not a pure or unique best nursing model that delivers the best care. Different features of different models can be adopted in the practice, so opting for a hybrid care delivery model, and can actually make the difference in producing an excellent patient experience. Top management chose the practice care delivery model, and the choice reflects its philosophy, values and economic conditions in which it operates ( Tiedeman and Lookinland, 2004 ). The aforementioned findings are the results of a research that focused on what is value in the patient perspective and aimed at detecting the features that have to be improved in order to enhance patients' excellent experience, by putting the nursing care organization in a more central place in the care planning, management and implementation ( Baret *et al.* , 2018 ). The findings of this study are expected to serve this purpose in rethinking, reorganizing and innovating healthcare services, by delivering a high quality nursing care. International experiences have highlighted how patients' responses have led to the promotion of innovations in nursing models ( Reeves and West, 2015 ), and PREMs are an excellent and powerful tool that can orient healthcare services' management and provision towards the patient-centered approach. Understanding and meeting patients' needs, to accordingly organize care, is a key strategy to improve healthcare services.

## Conclusions

Research has highlighted that providing high quality services is crucial and that user experience is key for measuring and improving the service quality, in particular in healthcare sector which is intensive in terms of human-human interactions. To the best of our knowledge, evidence on whether and how the organizational model of care service delivery can affect the patient experience is still at an initial stage. The results of this study reveal that healthcare services' uses appreciate those characteristics that are related to relation and interaction with a specific professional nurse. These features are identified as proxies of “ *be cared by* ,” of someone who is “ *in charge of* .” However, some of these features are judged by providers as not essential or professionalizing. Keeping them is essential for providing more user-centered services. Purely organizational features, mostly referring to the team organization, do not significantly or positively affect the patient experience. They seem transparent from the patient point of view and their effects should be additionally investigated.

Another key finding is that an excellent experience with the service has been found being positive affected by features of different models of delivery. We argue that service delivery models should not adopted as a whole, as pure models. Decision-makers and practitioners should mix features of different service delivery models into hybrid models, in order to better meet users' expectations.

## Figures and Tables

**Table 1:** Correlations among the nursing models' features and models

	F1	F2	F3	F4	F5	F6	F7	F8	Functional	Team nursing	Primary nursing/Case management
F1 Task-oriented work	1.0000										
F2 Nurse independent decision-making	0.1869*	1.0000									
	0.0000										
F3 Reference nurse	0.2852*	0.3111*	1.0000								
	0.0000	0.0000									
F4 Continuity after the acute event	−0.1552*	0.3063*	0.2486*	1.0000							
	0.0000	0.0000	0.0000								
F5 Caregiver's involvement	−0.2504*	−0.0621*	0.0482*	0.4871*	1.0000						
	0.0000	0.0000	0.0000	0.0000							
F6 Team of care	−0.1394*	0.2570*	0.1226*	0.6387*	0.4640*	1.0000					
	0.0000	0.0000	0.0000	0.0000	0.0000						
F7 Supervisor/team leader	−0.3134*	−0.1876*	−0.1566*	0.4417*	0.2592*	0.4425*	1.0000				
	0.0000	0.0000	0.0000	0.0000	0.0000	0.0000					
F8 Synergic team work	−0.3095*	0.1848*	0.0217*	0.5705*	0.5349*	0.8460*	0.4580*	1.0000			
	0.0000	0.0000	0.0359	0.0000	0.0000	0.0000	0.0000				
Functional	1.0000*	0.1869*	0.2852*	−0.1552*	−0.2504*	−0.1394*	−0.3134*	−0.3095*	1.0000		
	0.0000	0.0000	0.0000	0.0000	0.0000	0.0000	0.0000	0.0000			
Team nursing	−0.1956*	0.4280*	0.1592*	0.6879*	0.7038*	0.8784*	0.3484*	0.8913*	−0.1956*	1.0000	
	0.0000	0.0000	0.0000	0.0000	0.0000	0.0000	0.0000	0.0000	0.0000		
Primary nursing/Case management	−0.0067	0.5208*	0.5954*	0.7894*	0.2949*	0.5910*	0.5133*	0.5015*	−0.0067	0.6395*	1.0000
	0.5170	0.0000	0.0000	0.0000	0.0000	0.0000	0.0000	0.0000	0.5170	0.0000	

**Note(s):** For each variable, the first row includes the coefficients, where the star(*) indicates a statistical significance >0.05. The second row indicates the *p* value

**Table 2:** Linear logistic regression models results

	Human and relational aspects								Functional and technical aspects												Patient satisfaction			
	Emotional support						Relationship		Experienced competences and skills				Informative/Instrumental support											
	HP1		HP2		HP3		HP4		HP5		HP6		HP7		HP8		HP9		HP10		HP11		HP12	
	Fear and anxieties management by nurses		Nurse talk each other like the patient was not present		Respect and dignity by nurses		Patient involvement		Physician – nurse collaboration		Pain management		Communication with caregivers		Clear answers of nurses		Information at discharge – what to do once at home		Information at discharge – medications		Willingness to recommend		Overall assessment of the assistance	
	OR	*p*	OR	*p*	OR	*p*	OR	*p*	OR	*p*	OR	*p*	OR	*p*	OR	*p*	OR	*p*	OR	*p*	OR	*p*	OR	*p*
Patient responder	2.4	***	1.77	***	3.86	***	2.53	***	2.35	***	2.75	***	2.55	***	3.11	***	2.1	***	2.4	***	63.9	***	2.71	***
Male patient					1.22	***			1.21	***											1.25	**	1.1	*
Age	1.01	**	1.01	***	1.01	***	1.01	***	1.01	***	1.01	***	1.01	***	1.01	***	1.01	***	1.01	***	1.05	***	1.01	**
Educational level	0.87	***	0.83	***	0.82	***	0.83	***	0.92	***			0.83	***	0.78	***	−0.83	***			0.67	***		
State of health	1.53	***	1.26	***	1.62	***	1.03	***	1.67	***	1.57	***	1.09	***	1.53	***	1.59	***	1.66	***	2.11	***	1.86	***
Hospital access from ED	0.79	**			0.67	***	1.51	***	0.8	**	0.74	***	1.52	**	0.77	***	0.79	*	0.83	***	0.63	***	0.81	**
Task-oriented work (F1)	1.09	**			1.1	**			0.9	**									0.91	*				
Nurse independent decision-making (F2)			1.14	**	1.1	**			1.08	*							1.14	***	1.19	***				
Reference nurse (F3)	1.09	***			1.1	***											0.92	**					1.04	**
Continuity after the acute event (F4)	0.86	**			0.78	***									0.84	**					0.81	*	0.86	**
Caregiver's involvement (F5)	1.08	*					1.11	***																
Team of care (F6)																								
Supervisor/team leader (F7)					1.1	***									1.08	***					1.11	**		
Synergic team work (F8)																								

**Note(s):** ***, **, *Indicate level of significance at respectively *p* < 0.001, *p* < 0.01; *p* < 0.05

**Table 3:** Linear logistic regression models results

	Human and relational aspects								Functional and technical aspects												Patient satisfaction			
	Emotional support						Relationship		Experienced competences and skills				Informative/Instrumental support											
	Fear and anxieties management by nurses		Nurse talk each other like the patient was not present		Respect and dignity by nurses		Patient involvement		Physician – nurse collaboration		Pain management		Communication with caregivers		Clear answers of nurses		Information at discharge – what to do once at home		Information at discharge – medications		Willingness to recommend		Overall assessment of the assistance	
	OR	*p*	OR	*p*	OR	*p*	OR	*p*	OR	*p*	OR	*p*	OR	*p*	OR	*p*	OR	*p*	OR	*p*	OR	*p*	OR	*p*
Patient responder	2.4	***	1.77	***	3.86	***	2.53	***	2.35	***	2.75	***	2.55	***	3.11	***	2.1	***	2.4	***	63.9	***	2.71	***
Male patient					1.22	***			1.21	***											1.25	**	1.1	*
Age	1.01	**	1.01	***	1.01	***	1.01	***	1.01	***	1.01	***	1.01	***	1.01	***	1.01	***	1.01	***	1.05	***	1.01	**
Educational level	0.87	***	0.83	***	0.82	***	0.83	***	0.92	***			0.83	***	0.78	***	−0.83	***			0.67	***		
State of health	1.53	***	1.26	***	1.62	***	1.03	***	1.67	***	1.57	***	1.09	***	1.53	***	1.59	***	1.66	***	2.11	***	1.86	***
Hospital access from ED	0.79	**			0.67	***	1.51	***	0.8	**	0.74	***	1.52	**	0.77	***	0.79	*	0.83	***	0.63	***	0.81	**
Task-oriented work (F1)	1.09	**			1.1	**			0.9	**									0.91	*				
Nurse independent decision-making (F2)			1.14	**	1.1	**			1.08	*							1.14	***	1.19	***				
Reference nurse (F3)	1.09	***			1.1	***											0.92	**					1.04	**
Continuity after the acute event (F4)	0.86	**			0.78	***									0.84	**					0.81	*	0.86	**
Caregiver's involvement (F5)	1.08	*					1.11	***																
Team of care (F6)																								
Supervisor/team leader (F7)					1.1	***									1.08	***					1.11	**		
Synergic team work (F8)																								

**Note(s):** ***, **, *Indicate level of significance at respectively *p* < 0.001, *p* < 0.01; *p* < 0.05

**Table A1:** Key dimensions and aspects of the patient experience

Dimension	Aspect	Proxy
Technical aspects	Experienced competences and skills	Coordination and collaboration
		Pain management
	Informative/Instrumental support	Clarity of information
		Communication with caregivers
Human aspects	Emotional support	Fear and anxiety management
		Respect and dignity
	Relationship	Involvement in decision-making

**Table A2:** Summary of the main nursing models identified in literature, and of their key characteristics

Reference model	Features
Functional	Task-oriented work
Team nursing	Nurse independent decision-making
	Caregiver's involvement
	Team of care
	Synergic team work
Primary nursing/Case management	Supervisor/team leader
	Reference nurse
	Continuity after the acute event

**Table A3:** Questions used to assess the patient experience

	Dimension	Aspect	Question	Options of answer
Directly referred to nursing care delivery	Emotional support	Fear and anxiety management	Did nurses help you coping with fears and anxieties regarding your health condition during your hospital stay?	Always (5) – Often – Sometimes – Rarely – Never (1) – I did not experience fears and anxieties
		Respect and dignity	Did nurses talk to each other as you were not there during your hospital stay?	Always (5) – Often – Sometimes – Rarely – Never (1)
			Were you treated with dignity and respect by nurses during your hospital stay?	Always (5) – Often – Sometimes – Rarely – Never (1)
	Informative/Instrumental support	Clarity of information	Where the answers the nurses gave you during your hospital stay clear?	Always (5) – Often – Sometimes – Rarely – Never (1)
Generally referred to care delivered by hospitalists	Experienced competences and skills	Pain management	Do you think that the ward staff, during your hospital stay, did everything possible to help you managing pain?	I did not experience pain – Always (5) – Often – Sometimes – Rarely – Never (1)
	Relationship	Involvement in decision-making	Were you involved, during the hospital stay, in the decision-making process over your care path by the healthcare staff?	Always (5) – Often – Sometimes – Rarely – Never (1)
	Informative/Instrumental support	Communication with caregivers	Was it easy, during your hospital stay, for your family member or caregivers to receive information on your health conditions?	Always (5) – Often – Sometimes – Rarely – Never (1) – There was no need to – I was by myself and no family or caregivers were with me
	Experienced competences and skills	Coordination and collaboration	Ability to work together of medical and nursing ward staff: based on what you experienced during your hospital stay, how would you rate it?	Excellent (5) – Good – Acceptable – Weak – Poor (1)
	Informative/Instrumental support	Clarity of information	Did you receive clear information at discharge?	Yes, very clear (3) – Yes, clear enough – Not at all (1) – I did not receive any (1) – It was not necessary
	Satisfaction	Overall satisfaction	What is your overall assessment of the assistance received in the ward?	Excellent (5) – Good – Acceptable– Weak – Poor (1)
		Willingness-to-recommend	Would you advise the hospital to a relative or friend with a similar health problem?	Yes (3) – Perhaps – No (1)

**Table A4:** Summary of the features and their reference models.

Features	Description	Reference model
F1	Task-oriented work	Functional
F2	Nurse independent decision-making	Team nursing
F5	Caregiver's involvement	
F6	Team of care	
F8	Synergic team work	
F3	Reference nurse	Primary nursing/Case management
F4	Continuity after the acute event	
F7	Supervisor/team leader	

**Table A5:** Distribution of the nursing models in Tuscany Region.

Nursing model (Mean of their features – Table A6 )	Mean	SD	Min	Max
Functional	1.78	0.86	1	4
Team nursing	2.93	0.69	1.25	3.75
Primary nursing/Case management	2.83	0.58	1.5	3.75

**Note(s):** The original scale of evaluation ranges from 1 to 4, where 1 ≤ 30%, 2 = 30–50%, 3 = 50–70%, 4 ≥ 70%

**Table A6:** Distribution of each feature of the nursing models in Tuscany Region

Nursing model	Features	Description	1	2	3	4
			<30%	30–50%	50–70%	>70%
Functional	F1	Task-oriented work	48.33%	26.54%	23.25%	1.88%
Team nursing	F2	Nurse independent decision-making	11%	44.05%	32.34%	12.61%
	F5	Caregiver's involvement	16.14%	18.78%	31.42%	33.66%
	F6	Team of care	8.36%	4.95%	42.43%	44.26%
	F8	Synergic team work	9.46%	15.34%	19.21%	55.98%
Primary nursing/Case management	F3	Reference nurse	27.75%	22.11%	28.37%	21.77%
	F4	Continuity after the acute event	2.73%	9.36%	23.98%	63.93%
	F7	Supervisor/team leader	9.46%	15.34%	19.21%	55.98%

**Note(s):** Each item was evaluated in a scale from 1 to 4, where 1 ≤ 30%, 2 = 30–50%, 3 = 50–70%, 4 ≥ 70%

**Table A7:** Patients' experience with nursing practice from PREMs data

Variable (5–1 scale)	Mean	SD	Excellent and good ratings	Weak and poor ratings
Help in coping with fears and anxieties by nurses	4.30	1.01	83.4%	5.9%
The nurses did not talk to each other like the patient was not there	4.53	0.88	87.0%	4.0%
Respect and sense of dignity received by nurses	4.76	0.60	95.4%	1.5%
Clear answers received by nurses	4.62	0.73	92.6%	2.3%
Pain management	4.64	0.73	91.8%	2.5%
Involvement in care decisions	4.30	1.08	84.5%	7.8%
Difficulty in communicating with caregivers	4.54	0.86	87.7%	5.1%
Physician-nurse collaboration	4.44	0.75	92.2%	2.4%
Overall assessment of received assistance	4.53	0.69	89.7%	2.2%

Variable (3–1 scale)	Mean	SD	Excellent ratings	Poor ratings
Discharge information and instruction on what to control once home	2.7	0.51	70.4%	4.7%
Discharge information on medications	2.8	0.42	79.8%	2.2%
Would you advise the hospital? (Willingness-to-recommend *)*	2.86	0.4	94.0%	1.7%

**Table A8:** Description of the respondents to the PREMs survey

Socio-demographic characteristic	Numerosity	% Over total
*Age class*		
Under 30 y.o.	454	4.83%
30–49 y.o.	1,892	20.14%
50–59 y.o.	1,806	19.23%
60–69 y.o.	1,848	19.67%
70–79 y.o.	1,778	18.93%
Over 80 y.o.	1,616	17.20%
*Sex*		
Males patients	4,870	51.85%
Female patients	4,523	48.15%
*Educational level*		
Low	4,550	48.5%
Medium	3,350	35.7%
High	1,488	15.8%
*Perceived health status*		
Chronic patients	4,485	50.5%
Non chronic patients	4,402	49.5%
